# Leptin Drives Breast Cancer Aggressiveness Acting Through the Activation of the NCOA1/STAT3 Pathway

**DOI:** 10.3390/medsci14010032

**Published:** 2026-01-08

**Authors:** Khouloud Ayed, Amal Gorrab, Hichem Bouguerra, Rym Akrout, Sami Zekri, Wassim Y. Almawi, Rahma Boughriba, Khalil Choukri, Dhouha Bacha, Alessandra Pagano, Jean-François Louet, Hervé Kovacic, Mounia Tannour-Louet, Asma Gati

**Affiliations:** 1 Laboratory of Genetics, Immunology, and Human Pathologies, Department of Biology, Faculty of Sciences of Tunis, University Tunis El Manar, Tunis 2092, Tunisia; khouloud.ayed@etudiant-fst.utm.tn (K.A.);; 2 Confocal Microscopy Unit, Faculty of Medicine of Tunis, University Tunis El Manar, Tunis 1007, Tunisia; 3 Faculty of Sciences of Tunis, University Tunis El Manar, Tunis 2092, Tunisia; 4 Faculty of Pharmacy, University of Monastir, Monastir 5000, Tunisia; 5 Service d’Anatomie Pathologique, Hôpital Mongi Slim, La Marsa 2046, Tunisia; 6 Equipe 9 Cytosquelette et Neurophysiopathologie, Faculté Pharmacie site Timone, UMR7051 Institut de Neuro Physiopathologie (INP), 27 Bd Jean-Moulin, 13005 Marseille, France; 7 Adipo-Cible Research Study Group, Mediterranean Center of Molecular Medicine, Department of Molecular and Cellular Biology, Côte d’Azur University Inserm, 06204 Nice, France; 8 CNRS, Inserm, Institute of Molecular and Cellular Pharmacology, Côte d’Azur University, 06560 Valbonne, France

**Keywords:** breast cancer, leptin, obesity, tumor migration, epithelial–mesenchymal transition

## Abstract

**Background/Objectives**: Obesity-associated hyperleptinemia has been linked to breast cancer (BC) progression via mechanisms that remain incompletely understood. This study explores the role of leptin and its receptor (LEPR) in facilitating BC cell proliferation, migration, epithelial–mesenchymal transition (EMT), and STAT3 signaling pathway activation. **Methods**: We analyzed gene expression and survival data from TCGA BRCA dataset. MCF-7 and MDA-MB-231 BC cells were exposed to leptin at 10 ng/mL (lean-associated levels) and 100 ng/mL (elevated levels linked to obesity). MTT assays, colony formation tests, wound-healing and tumor spheroid dissemination experiments evaluated cell proliferation and migration. Immunofluorescence and Western blot analysis assessed changes in EMT markers and cytoskeletal alterations, while Western blotting and qPCR assessed STAT3 and *NCOA1* expression and activation levels. **Results**: Elevated *LEPR* expression was linked with unfavorable prognosis in BC patients. Higher doses of leptin (100 ng/mL) significantly enhanced cellular proliferation rates and migratory capabilities, in both cell lines, and promoted EMT characteristics marked by downregulated E-cadherin and cytoskeleton structural changes. Whereas heightened JAK2/STAT3 signaling correlated with elevated leptin dosages, STAT3 inhibition using AG490 reversed leptin-induced migration while reinstating E-cadherin levels to baseline. Furthermore, leptin upregulated *NCOA1*, an essential STAT3 coactivator, facilitating increased expression of *Cyclin D1* and *VEGF* target genes. Clinical positive relationships were seen between *LEP*/*LEPR* expressions and *NCOA1* levels and between *NCOA1* and various gene signatures related to STAT3/P-STAT3 within BC specimens. **Conclusions**: Obesity-associated hyperleptinemia enhances aggressiveness in BC through a mechanism involving LEPR-mediated activation pathways encompassing NCOA1/STAT3, which drive proliferation, migration, and EMT. This assigns a potential therapeutic utility for obesity-related advancements found within BC pathology.

## 1. Introduction

Breast cancer (BC) remains the most commonly diagnosed malignancy in women globally. Despite notable progress in diagnostic techniques and therapeutic interventions, BC continues to rank as the second leading cause of cancer-related mortality [1]. Although the minority (3%) of BC cases can be traced back to genetic predisposition, exemplified by hereditary mutations, notably in the *BRCA1* and *BRCA2* tumor suppressor genes, most BC cases are linked to environmental factors and lifestyle choices. Among these, obesity stands out as a significant contributor [2,3,4]. Earlier meta-analyses reported an increased body mass index (BMI) of 5 kg/m^2^ was parallel with a 12% rise in BC risk. Furthermore, women with BC and obesity present with 30–40% higher mortality risk than their lean counterparts [5,6]. While substantial epidemiological evidence linked obesity with increased incidence of BC, the precise molecular mechanism underlying this relationship remains incomplete.

Several studies revealed that altered adipokine levels linked with obesity are crucial to BC progression [7,8]. Among these adipokines, leptin, a 16 kDa peptide hormone encoded by the *Ob* gene, has gained considerable attention as a major regulatory contributor. Leptin is synthesized by adipocytes in response to overall fat mass and is also released from other sources such as the stomach, skeletal muscle, placenta, and cancer cells [9]. Owing to its role in regulating energy homeostasis and food intake [10], leptin mediates its molecular actions through binding to its transmembrane receptor (LEPR). Among the different isoforms, the long form LEPRb possesses the full-length intracellular domain required for optimal signaling activity [10]. Various intracellular signaling pathways are triggered upon leptin engaging its receptor, including phosphatidylinositol-3-kinase (PI3K), mitogen-activated protein kinase (MAPK), and Janus kinase 2/signal transducer and activator of transcription 3 (JAK2/STAT3) [11]. In turn, the activation of these pathways influences critical cellular processes including survival, proliferation, differentiation, invasion, and migration [12].

This study investigates how leptin signaling contributes to BC aggressiveness and explores the underlying molecular mechanisms involved. Our results indicated that elevated leptin levels, reflecting hyperleptinemia seen in obesity, promote cellular proliferation, migration and epithelial–mesenchymal transition (EMT) in BC cells. These effects occur through STAT3 activation and enhanced expression of the NCOA1 nuclear coactivator. These findings further confirm the role of leptin in advancing BC and highlight its relevance as a therapeutic target for obesity-related BC.

## 2. Materials and Methods

### 2.1. Bioinformatics Analyses

The Breast Invasive Carcinoma (TCGA, PanCancer Atlas) dataset, including 1081 primary BC cases, was used in this study, and RNA sequencing data were retrieved from cBioportal (https://www.cbioportal.org/ (accessed on 6 March 2024)). The Gene Expression Profiling Interactive Analysis (GEPIA) database (http://gepia.cancer-pku.cn (accessed on 12 September 2024)) investigated the *LEPR* mRNA expression level across various molecular subtypes of BC. The prognostic significance of *LEPR* was assessed through overall survival (OS) analyses, in which BC cases were divided into high- and low-expression groups according to the lower quartile thresholds for *LEPR* expression. A logrank *p*-value set at <0.05 was considered statistically significant.

A validated scoring system based on transcriptomic analysis and comprising 16 genes was used to determine the EMT SCORE, as described [13]: EMT SCORE = *VIM* + *CDH2* + *FOXC2* + *SNAI1* + *SNAI2* + *TWIST1* + *FN1* + *ITGB6* + *MMP2* + *MMP3* + *MMP9* + *SOX10* + *GCS* − *CDH1* − *DSP* − *OCLN*. In addition, gene signatures of STAT3 and its phosphorylated form, P-STAT3, were analyzed within the Breast Invasive Carcinoma dataset from TCGA (PanCancer Atlas), following established methodologies [14,15]. Furthermore, protein–protein interaction (PPI) networks for LEP, LEPR, NCOA1, and STAT3 were created using Gene Multiple Association Network Integration Algorithm (GeneMANIA; http://www.genemania.org/ (accessed on 14 September 2024)).

### 2.2. Cell Culture, Treatments, Adenoviral Transduction

The human BC cell lines MCF-7 (RRID:CVCL_0031) and MDA-MB-231 (RRID:CVCL_0062) were obtained from American Type Cell Collection (ATCC, Manassas, VA, USA). MCF-7 were cultured in RPMI, while MDA-MB-231 were maintained in DMEM. Both media were supplemented with 10% fetal bovine serum, 100 U/mL penicillin, 100 mg/mL streptomycin, and 1% sodium pyruvate (all obtained from ThermoFisher Scientific, Waltham, MA, USA). Cells were seeded at 10^6^ cells/mL and incubated at 37 °C in a humidified atmosphere containing 5% CO_2_.

For leptin experiments, the cells were treated in serum-free media with human recombinant leptin (Sigma-Aldrich, Darmstadt, Germany) at 10 ng/mL or 100 ng/mL. In inhibition analysis experiments, the cells were pretreated with the STAT3 inhibitor, AG490, at 100 μM for 45 min before being treated with leptin. In addition, NCOA1 adenoviruses were employed for adenoviral transduction as previously outlined [16].

### 2.3. Flow Cytometry

MCF-7 and MDA-MB-231 cells were subjected to trypsinization, followed by two washes with cold PBS. The cells were then incubated with 5 μL of FITC-conjugated monoclonal anti-LEPR antibody (R&D Systems; Minneapolis, MN, USA) for 30 min at 4 °C. Following incubation, excess antibody was washed out with PBS, and fluorescence levels were determined on Accuri C6 flow cytometer (BD Biosciences, Dubai, United Arab Emirates).

### 2.4. Cell Proliferation Assays

Cell proliferation was assessed using the MTT assay. Specifically, 5 × 10^3^ cells were plated in a 96-well flat-bottom microtiter plate and incubated with leptin at 10 ng/mL or 100 ng/mL for 48 h. Following the incubation period, the culture media were aspirated, and the cells were exposed to MTT (0.5 mg/mL) for four hours at 37 °C. The formed formazan crystals were then dissolved in DMSO, and absorbance was measured at 570 nm using a microplate reader.

### 2.5. Colony Formation Assay

MCF-7 and MDA-MB-231 cells were seeded in 6-well plates at 2500 cells/well overnight and treated with leptin at 10 ng/mL or 100 ng/mL for 48 h. The culture media were subsequently refreshed every two days over a 14-day period. Subsequently, the media was removed, and the colonies were carefully washed with PBS, fixed with cold methanol, and stained with 0.5% crystal violet.

### 2.6. Wound-Healing Assay

A wound-healing assay evaluated cell migration. MCF-7 and MDA-MB-231 cells (4 × 10^5^ cells/well) were cultured in 6-well plates to form a confluent monolayer. Scratches were created using a micropipette tip, and the scratch closure was monitored by phase-contrast microscopy and captured at 0 and 48 h. The migration of cells was assessed using ImageJ software, version 1.52v.

### 2.7. Tumor Spheroid Dissemination Assay

MCF-7 and MDA-MB-231 cell spheroids were formed based on the hanging drop method. Briefly, 20 μL droplets of cell-containing culture medium with 2000 cells were placed into the lid. The lid was then inverted onto the base of a Petri dish containing 5 mL of PBS. After 5 days of culture, spheroids were transferred to a 24-well plate and treated with leptin. Tumor spheroid dissemination was monitored and photographed at 0 and 48 h. Quantification was performed using the Image J software.

### 2.8. Immunofluorescence and Confocal Imaging

MCF-7 cells (4 × 10^4^ cells/well) were cultured in a Lab-Tek II chamber slide (Thermo Fisher Scientific, Waltham, MA, USA) and treated with leptin (100 ng/mL) and AG490 (100 µM). The cells were fixed with 4% formaldehyde solution, followed by permeabilization with PBS containing 0.1% Triton X-100, and then blocked with PBS solution containing 1% BSA. Cells were incubated with anti-E-cadherin (ab15148) primary antibody (at 1:100) overnight at 4 °C, followed by the secondary anti-rabbit IgG Alexa Fluor 488 antibody (at 1:1000) for 45 min at room temperature under light-tight conditions. Phalloidin-iFluor 488 conjugate (ab176753) was applied at 1:1000 concentration, followed by an additional incubation for 30 min at room temperature. Fluorescence signals were analyzed using a Zeiss Axio Observer 7-Apotome 3 confocal microscope (Zeiss, Hanover, Germany).

### 2.9. Western Blot

MCF-7 and MDA-MB-231 cells were treated with leptin at 0 (vehicle control), 10 ng/mL, and 100 ng/mL. Cellular proteins were extracted using a cell lysis buffer supplemented with protease and phosphatase inhibitors. After centrifugation for 20 min at 12,000 rpm at 4 °C to remove the cellular debris, supernatants were separated on 10% SDS-PAGE gel. Separated proteins were transferred to nitrocellulose membranes (Roche, Indianapolis, IN, USA), which were blocked with 5% dry milk in Tris-buffered saline/0.1% Tween 20. The membranes were incubated with primary antibodies to STAT3, P-STAT3, and β-Actin (at 1:5000). They were then washed (3X) with TBST buffer (20 mM Tris–HCl, pH 7.5, 137 mM NaCl, and 0.05% Tween 20). Subsequently, the membranes were incubated with either anti-rabbit peroxidase-conjugated secondary antibody (for STAT3 at 1:5000), or anti-mouse peroxidase-conjugated (for P-STAT3, and β-Actin at 1:1000) secondary antibody, for 1 h at room temperature. Protein bands were detected with Amersham ECL detection reagent (Amersham Pharmacia Biotech, Wilmington, DE, USA). Densitometric analysis of bands was performed using ImageJ software and normalized to β-actin as a loading control.

### 2.10. RNA Analysis

Total cellular RNA was extracted using the RNeasy Mini kit (Qiagen, Hilden, Germany), and the reverse transcription was done using the Superscript III kit (Invitrogen, ThermoFisher Scientific, Waltham, MA, USA). Quantitative PCR (qPCR) was performed using sequence-specific primers obtained through Roche (Universal Probe Library). The relative fold change in expression levels for *Cyclin D1*, *VEGF*, *STAT3*, *NCOA1*, *ERα*, *ERRα*, and *ERRγ* was normalized against the housekeeping gene, *GAPDH*. The primer sequences used for qRT-PCR are shown in Table S1.

### 2.11. Statistical Analysis

All experiments were performed independently at least three times. The results were expressed as mean ± standard deviation (SD). Statistical analysis was performed using Graph-Pad Prism software, version 8.0.1 (GraphPad Software, Inc., La Jolla, CA, USA). The significance of the differences between two groups was assessed using Student’s *t*-test, while comparisons between multiple groups were made using one-way ANOVA followed by post hoc multiple comparisons test. Pearson’s correlation coefficient was used to evaluate the relationship between two variables. A *p* value < 0.05 was considered statistically significant.

## 3. Results

### 3.1. LEPR Expression Correlates with Adverse Prognosis in Breast Cancer

**Figure 1:**
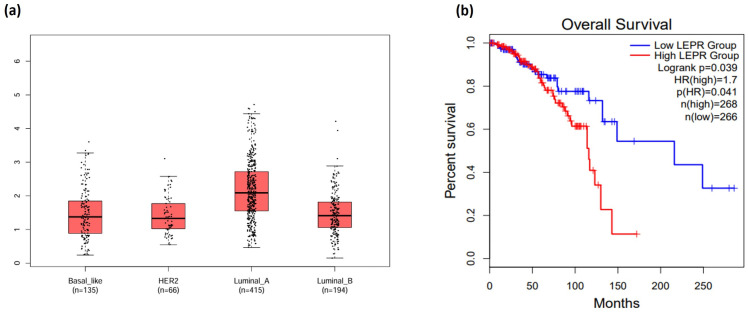
*LEPR* is expressed by human BC tumors and correlates with poor BC prognosis. (**a**) Box-whisker plots generated by GEPIA showing *LEPR* mRNA expression levels across BC molecular subtypes. (**b**) Kaplan–Meier survival curve based on GEPIA database revealing significantly shorter overall survival in BC patients with higher *LEPR* expression (group cutoff = quartile).

An analysis of *LEPR* mRNA expression across various molecular subtypes of BC using GEPIA was conducted on 1080 human BC tumors from The Breast Invasive Carcinoma (TCGA, PanCancer Atlas). Results obtained showed that *LEPR* mRNA was present in all four major human BC molecular subtypes: basal-like, HER2-enriched, luminal A, and luminal B (Figure 1a). Survival analyses demonstrated a significant association between elevated *LEPR* expression and reduced overall survival rates among BC patients (Log-rank *p* = 0.039; Figure 1b). These results indicate that increased *LEPR* expression is a contributor to poor clinical outcome in BC patients.

### 3.2. Elevated Leptin Levels Promote Breast Cancer Cells Proliferation

**Figure 2:**
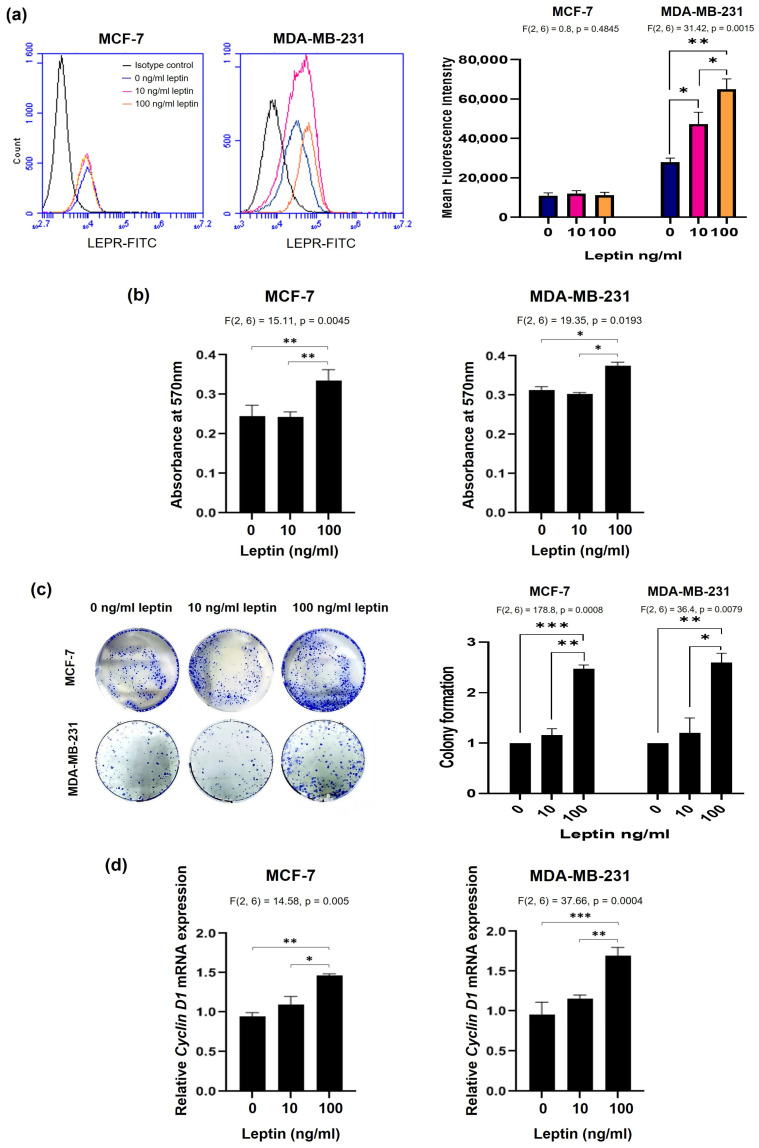
A high leptin dose, mimicking hyperleptinemia in individuals with obesity, is exclusively able to enhance BC cells proliferation. (**a**) LEPR expression levels in MCF-7 and MDA-MB-231 cells were quantified by flow cytometry, and the effect of leptin (10 and 100 ng/mL) on LEPR expression in both cell lines was assessed after 48 h. Mean fluorescence intensity (MFI) was quantified from flow cytometry data. (**b**) MCF-7 and MDA-MB-231 proliferation was assessed after 48 h treatment with 10 or 100 ng/mL of leptin using an MTT assay. (**c**) Colony formation ability of MCF-7 and MDA-MB-231 cells was evaluated after 14 days of culture. Colony area was quantified, and data are presented as fold change relative to the control. (**d**) *Cyclin D1* expression levels in MCF-7 and MDA-MB-231 cells were determined by qRT-PCR after treatment with 10 or 100 ng/mL of leptin for 48 h. Data were normalized to endogenous *GAPDH* mRNA levels. * *p* < 0.05; ** *p* < 0.01; *** *p* < 0.001.

Flow cytometry demonstrated the expression of LEPR on both MCF-7 and MDA-MB-231 BC cell surfaces (Figure 2a). The treatment of these two cell lines with leptin at 10 ng/mL and 100 ng/mL, concentrations that mimic plasma leptin levels in lean individuals and those with obesity [17], led to marked overexpression of LEPR in (the more aggressive cell line) MDA-MB-231 cells but not in MCF-7 cells. 

We also tested the effect of leptin on the proliferation of MCF-7 and MDA-MB-231 BC cells using an MTT assay and a colony formation assay by treating the cells with leptin at 10 ng/mL and 100 ng/mL concentrations. Treatment with leptin at 100 ng/mL, but not 10 ng/mL, stimulated the proliferation of both MCF-7 (*p* = 0.0078) and MDA-MB-231 (*p* = 0.0368) cells (Figure 2b). Similarly, colony formation assays revealed that treatment with leptin at 100 ng/mL stimulated colony growth; no effects were seen at 10 ng/mL (Figure 2c). Furthermore, *Cyclin D1*, an essential growth sensor involved in cell cycle progression, was markedly upregulated in cells treated with leptin at 100 ng/mL (*p* = 0.0004 for MCF-7; *p* = 0.001 for MDA-MB-231). In contrast, treatment with 10 ng/mL did not affect *Cyclin D1* expression (Figure 2d).

### 3.3. High Leptin Levels Stimulate BC Cell Migration and EMT

**Figure 3:**
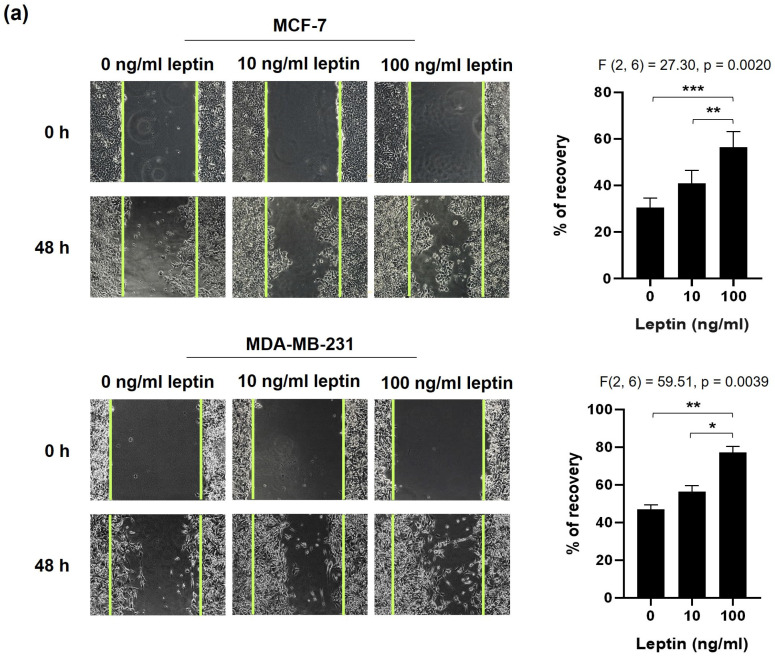

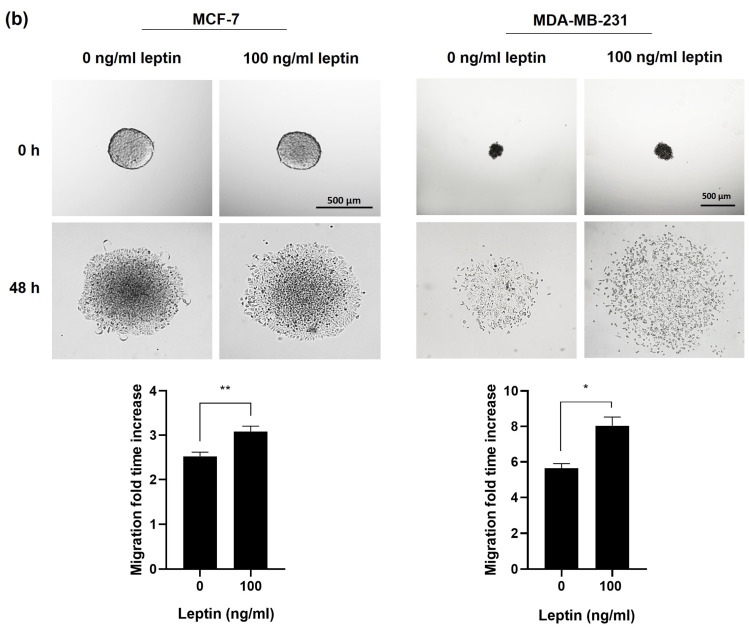
Elevated leptin levels promote BC cell 2D and 3D migration. MCF-7 and MDA-MB-231 cells were treated with 10 or 100 ng/mL of leptin and subjected to (**a**) wound-healing and (**b**) spheroid dissemination assays. Images were captured at 0 h and 48 h post-treatment. Cell migration was quantified. * *p* < 0.05; ** *p* < 0.01; *** *p* < 0.001.

The wound-healing assay revealed that treatment with 100 ng/mL leptin markedly enhanced the migration of MCF-7 (*p* = 0.002) and MDA-MB-231 (*p* = 0.0192) cells compared to both untreated controls and cells treated with leptin at 10 ng/mL (*p* < 0.0001 for each; Figure 3a). These findings support the pro-migratory effect of elevated leptin levels, commonly observed in patients with BC and obesity. This effect was further supported by spheroid dissemination assay, which showed increased cell spreading from spheroids of both cell lines following treatment with 100 ng/mL leptin (*p* = 0.0093 for MCF-7 and *p* = 0.0261 for MDA-MB-231; Figure 3b).

**Figure 4:**
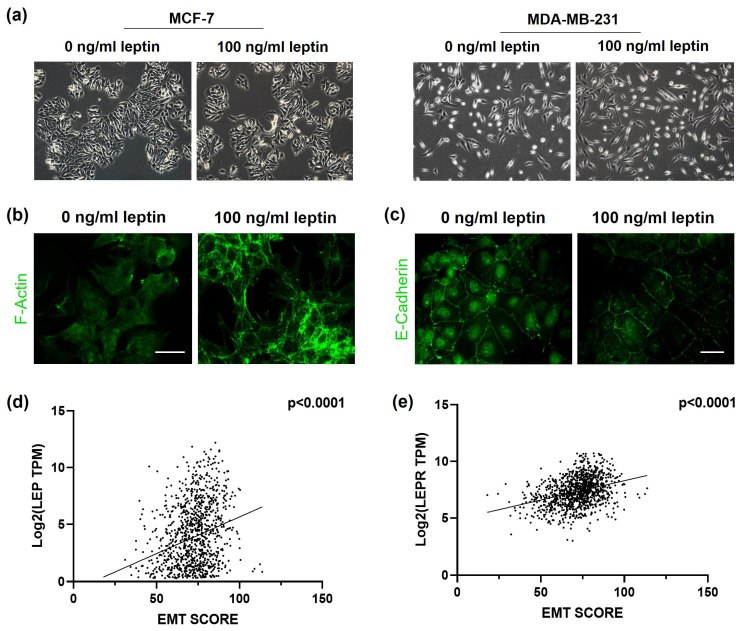
High leptin levels stimulate EMT in BC cells. (**a**) Representative phase-contrast microscopy images illustrating morphological changes in MCF-7 and MDA-MB-231 cells following 48 h treatment with 100 ng/mL of leptin. (**b**) Representative confocal microscopy images of F-Actin immunofluorescence in MCF-7 cells treated with 100 ng/mL leptin for 48 h. (**c**) Representative confocal microscopy images of E-Cadherin immunofluorescence in MCF-7 cells treated with 100 ng/mL leptin for 48 h. Scale bar: 20 μm. Scatter plots showing correlation of EMT SCORE with (**d**) *LEP* and (**e**) *LEPR* expression in The Breast Invasive Carcinoma (TCGA, PanCancer Atlas) dataset.

Morphological examinations demonstrated that exposure to 100 ng/mL of leptin induced a mesenchymal-like phenotype in both cell lines, evidenced by the elongated, spindle-shaped morphology and disrupted cell–cell junction (Figure 4a). Confocal microscopy using FITC-conjugated phalloidin staining demonstrated an altered F-actin cytoskeleton, revealing reorganization into thick linear stress fibers in the 100 ng/mL leptin-treated MCF-7 cells (Figure 4b). Furthermore, the levels of the epithelial marker, E-cadherin, were significantly diminished in MCF-7 cells treated with leptin at 100 ng/mL (Figure 4c). Consistent with these cellular findings, EMT scores derived from data on breast cancer patients exhibited a robust positive correlation with *LEP* and *LEPR* expression levels (*p* < 0.0001; Figure 4d,e). This indicated that high (100 ng/mL) leptin levels were more efficient than low (10 ng/mL) levels in promoting migration and inducing EMT in BC cells.

### 3.4. Elevated Leptin Stimulates STAT3 Signaling in Breast Cancer Cells

**Figure 5:**
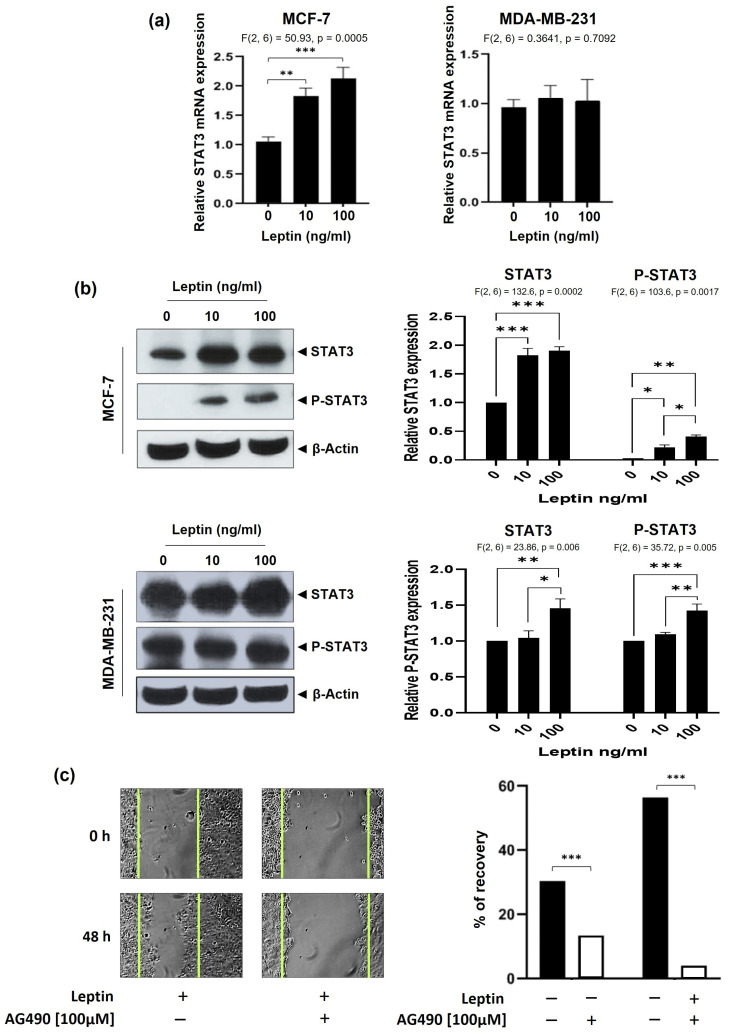

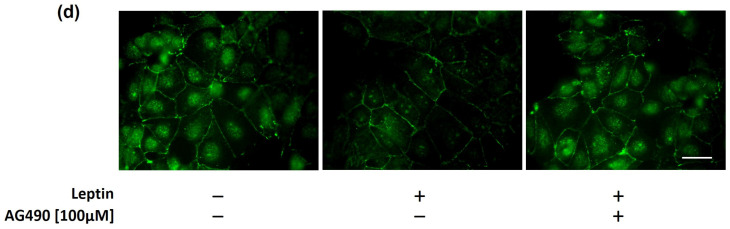
Elevated leptin dose triggers increased STAT3 expression and phosphorylation. (**a**) *STAT3* gene expression levels in MCF-7 and MDA-MB-231 cells were assessed by qRT-PCR following 24 h treatment with 10 or 100 ng/mL of leptin. (**b**) Representative Western blot showing total STAT3 and phosphorylated STAT3 protein levels in MCF-7 and MDA-MB-231 cells treated with different leptin concentrations (10 and 100 ng/mL) for 24 h. Band intensities were quantified using ImageJ software and normalized to β-actin levels. (**c**) MCF-7 were subjected to wound healing assay following treatment with 100 ng/mL of leptin, with or without 100 µM of AG490 (STAT3 inhibitor). Images were captured at 0 h and 48 h post-treatment. Cell migration was quantified, and the recovery percentage was calculated. (**d**) Representative confocal microscopy images of E-Cadherin immunofluorescence in MCF-7 cells treated with 100 ng/mL of leptin, in the presence or absence of AG490 (100 µM). Scale bar: 20 μm. * *p* < 0.05; ** *p* < 0.01; *** *p* < 0.001.

Western blotting showed a significant increase in total STAT3 and phosphorylated STAT3 (P-STAT3) levels after treating MCF-7 and MDA-MB-231 cells with 100 ng/mL leptin (Figure 5b). The activation of STAT3 was more pronounced at the 100 ng/mL than the 10 ng/mL concentration, indicating a dose-dependent relationship. The role of STAT3 in the leptin-driven migration of BC cells was further investigated by pre-treating MCF-7 cells with the STAT3 inhibitor, AG490, before treatment with leptin at 100 ng/mL. Results obtained revealed that AG490-induced inhibition impeded leptin-stimulated cell migration (Figure 5c) and restored E-cadherin expression (Figure 5d), thus reinforcing the involvement of STAT3 in promoting the pro-migratory effects induced by leptin.

### 3.5. Leptin Regulates NCOA1 Expression, Enhancing STAT3 Transcriptional Activity

**Figure 6:**
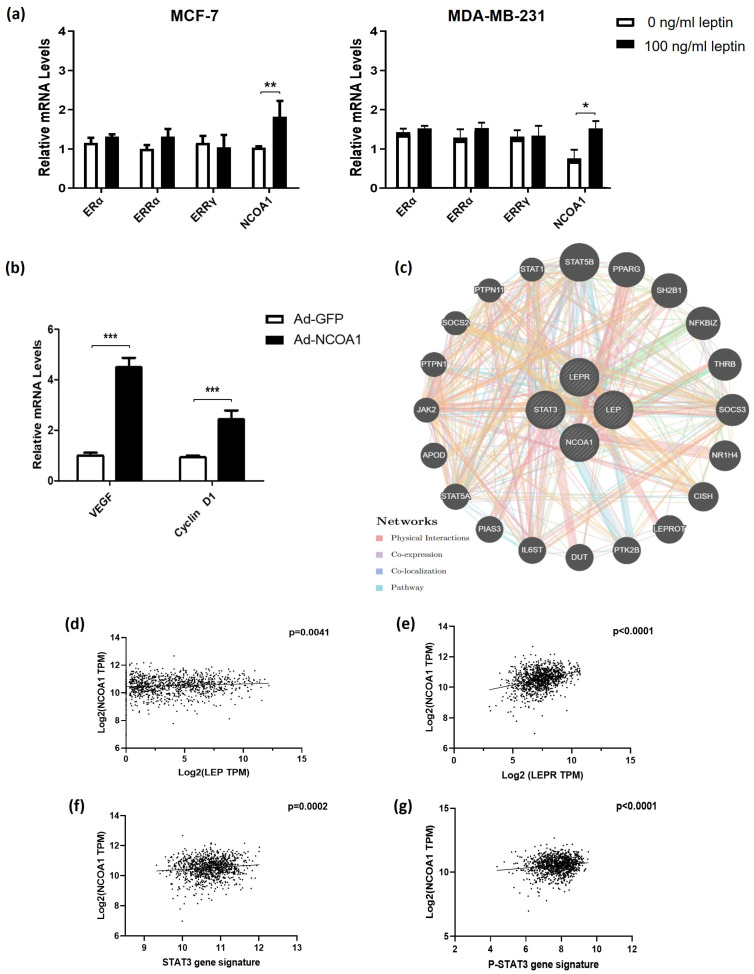
Increased expression of nuclear coactivator 1 (*NCOA1*) was induced by elevated leptin doses and was found to be positively correlated with *LEP*, *LEPR*, and STAT3 target gene expression in BC patients. (**a**) *ERα*, *ERRα*, *ERRγ*, and *NCOA1* gene expression levels in MCF-7 and MDA-MB-231 cells were assessed by qRT-PCR following treatment with 100 ng/mL of leptin for 24 h. Data were normalized to endogenous *GAPDH* mRNA levels. (**b**) MCF-7 cells were transduced with Ad-NCOA1, and 48 h later, mRNA levels of 2 STAT3 target genes, *VEGF* and *Cyclin D1*, were measured by qRT-PCR. * *p* < 0.05; ** *p* < 0.01; *** *p* < 0.001. (**c**) Protein–protein interaction (PPI) network of LEP, LEPR, NCOA1, STAT3, and related genes. GeneMANIA plot revealing that these genes were linked by physical interaction at 77.64%. Scatter plots showing correlation of *NCOA1* with (**d**) *LEP* and (**e**) *LEPR* expression in The Breast Invasive Carcinoma TCGA PanCancer Atlas dataset. Correlation of *NCOA1* with (**f**) STAT3-gene signature and (**g**) P-STAT3-gene signature in the same dataset. Pearson’s correlation coefficient was applied to assess the association between variables, with *p* values < 0.05 considered statistically significant.

We investigated the effect of elevated leptin on the expression of transcriptional factors that modulate STAT3 activity and downstream target genes by examining *NCOA1*, *ERα*, *ERRα*, and *ERRγ* expression levels in MCF-7 and MDA-MB 231 cells following leptin treatment. A significant upregulation of only the STAT3 co-activator, *NCOA1*, was noted in both cell lines treated with 100 ng/mL of leptin (Figure 6a). This overexpression of *NCOA1* in MCF-7 cells resulted in heightened expression of *Cyclin D1* and *VEGF*, two established target genes regulated by STAT3 in several cancer types (Figure 6b). This was further supported by patient data, which demonstrated strong positive correlations between *LEP* (*p* = 0.0041) and *LEPR* (*p* < 0.0001) expressions and *NCOA1* levels (Figure 6d,e). In addition, notable associations were seen between *NCOA1* and the gene signatures for STAT3 and P-STAT3 (*p* = 0.0002; *p* < 0.0001, respectively; Figure 6f,g).

## 4. Discussion

Several epidemiological studies reported that obesity is associated with an increased risk of BC recurrence and related death, independent of menopause status [18,19,20,21]. While the relationship between obesity and BC pathogenesis is well-established, the molecular mechanisms underlying this association remain incomplete. Here, we demonstrate that leptin signaling is key to BC progression, particularly in the context of obesity. Indeed, elevated leptin levels, a surrogate of hyperleptinemia seen in individuals with obesity, markedly enhance the proliferation, migration, and EMT of BC cells through STAT3 activation and *NCOA1* upregulation. These findings underscore the significant contribution of the Leptin/LEPR/NCOA1/STAT3 axis to BC aggressiveness and highlight its potential as a therapeutic target for obesity-related BC [22].

Given their unique molecular profiles and phenotypic traits, the MCF-7 and MDA-MB-231 cell lines were used as in vitro models for investigating the link between obesity and BC progression [23]. The MCF-7 cells signify hormone receptor-positive (ER+/PR+) BC, sensitive to estrogen and metabolic components, including adipokine signaling [19]. This renders MCF-7 cells instrumental in investigating hormonal effects on obesity, such as leptin, through modulation of estrogen receptor signaling pathways and tumor growth [23,24]. On the other hand, the MDA-MB-231 cells represent TNBC, an aggressive metastatic BC subtype, characterized by deficient ER, PR, or HER2 expression [25]. Considering the correlation between obesity and poor clinical outcomes, these cells serve as a valuable model for investigating how obesity enhances tumor invasiveness, EMT, and metastasis and allow for comprehension of leptin’s involvement in tumor development [23].

While a strong epidemiological connection was made between obesity and increased risk for BC, the molecular mechanisms are not fully elucidated [8,26]. Earlier studies demonstrated that leptin supports cancer progression by modulating various carcinogenesis mechanisms, including cell cycle regulation, secretion of angiogenic proteins, and enhancing migration [11,27,28,29,30]. By promoting the fatty acid oxidation pathway, we recently showed that leptin enhances BC cell resistance to NK lysis by upregulating the peroxisome proliferator-activated receptor coactivator-1α (PGC1A) [31]. Here, we showed that *LEPR* mRNA was expressed in human BC tissues, regardless of molecular subtype. Moreover, high *LEPR* expression was correlated with poor prognosis among BC patients. This finding aligns with earlier reports suggesting that LEPR overexpression facilitates tumor advancement in several cancer types [32], including triple-negative BC [33], prostate cancer [34], and endometrial cancer [35]. Survival analysis further indicates that high *LEPR* levels are associated with diminished overall survival rates, reinforcing the need to further explore LEPR expression and leptin signaling as a prognostic indicator for BC and other malignancies [32].

The association of leptin with enhanced BC cell proliferation remains controversial. While some in vitro studies have reported that leptin stimulates BC cell proliferation [36,37,38,39,40,41], others have shown that leptin does not affect the proliferation of BC cell lines [42,43]. To further establish leptin’s role in obesity-induced BC progression, we compared the effects of a high leptin dose (100 ng/mL), which mimics leptin plasma levels in individuals with obesity [13], with those of a low dose (10 ng/mL), commonly seen in lean individuals [17], on the activities of MCF-7 and MDA-MB-231 cells. Exposure to 100 ng/mL rather than 10 ng/mL leptin concentrations significantly boosts BC cells’ proliferation and colony formation, indicating a dose-dependent relationship [44]. The increases in *Cyclin D1* at 100 ng/mL concentrations further emphasize leptin’s role in regulating cell cycle progression, promoting cellular proliferation, and fostering aggressive phenotypes characteristic of obesity-driven cases [44,45,46].

Consistent with observations demonstrating the capacity of leptin to promote invasiveness in various cancers, the wound-healing and tumor spheroid dissemination assays used here further validate leptin’s role in promoting migratory behavior in BC cells [12]. At a 100 ng/mL concentration, leptin promoted the migratory capacity of MCF-7 and MDA-MB-231 cells. Similar findings have been reported in other BC cell lines treated with high leptin doses, including MDA-MB-468, HCC-1806, and T47D, suggesting a pivotal role for leptin in BC progression in women with obesity [47,48]. The E-cadherin downregulation confirmed the efficacy of high doses of leptin-inducing EMT and the initiation of morphological changes and actin cytoskeleton remodeling, consistent with a transition from an epithelial to a mesenchymal status [49,50,51]. This was reminiscent of the findings of Yan et al., who reported that 100 ng/mL leptin stimulation augmented fibronectin, N-cadherin, and vimentin expression but reduced E-cadherin and occluding expression in MCF-7 cells [52]. Leptin was also found to regulate the expression of EMT-associated genes, including MMP-7, MMP-9, vimentin, ZEB-1 and Twist, further supporting its involvement in EMT induction [52,53].

A key finding of this study is that leptin drives EMT via STAT3 activation, a central event in cancer metastasis, evidenced by decreased E-cadherin levels and cytoskeletal alterations. This is consistent with leptin’s reported ability to activate multiple signaling pathways, in particular the JAK2/STAT3 pathway, which is involved in distinct steps of carcinogenesis, including proliferation, apoptosis, migration, and angiogenesis [54,55,56,57,58]. This pathway is also involved in EMT by enhancing the extracellular matrix protein synthesis, such as MMP-2 [59,60] and MMP-13 [60]. In addition, it induces the expression of EMT-inducing transcription factors (EMT-TFs), including Snail, Zeb1, JUNB, and Twist-1 [61]. Other signaling pathways may also be involved in leptin-induced EMT, particularly the upregulation of PKM2 expression and the activation of PI3K/AKT [62] and ERK signaling pathways [63].

Moreover, inhibiting the JAK2/STAT3 signaling pathway using AG490 effectively curtailed migration induced by leptin while restoring E-cadherin expression level, further confirming its role as a key mediator in leptin’s pro-migratory properties [64]. This corroborates existing literature asserting STAT3’s contribution towards migratory capabilities amongst tumor cells [58,59,60,61,62,63,64,65], affirming its significance concerning progressions related specifically to obesity-associated breast malignancies. It should nevertheless be acknowledged that AG490 lacks complete specificity toward STAT3, as it may also inhibit upstream kinases such as JAK2, JAK3, and EGFR. Therefore, our conclusions do not solely attribute the observed effects to STAT3 inhibition and do not exclude potential contributions from these upstream signaling components.

Another key finding of this study is the upregulation of *NCOA1* upon treatment with leptin. NCOA1 is a coactivator for several STAT family members, including STAT6 and STAT5 [66,67,68]. It has also been reported to associate with the transactivation domain of STAT3, thereby enhancing the transcription of STAT3 target genes, such as *Cyclin D1*, *VEGF*, *c-myc*, *p21waf1*, *Bcl2*, *Bcl-xL*, and *β2-macroglobulin* [67,69,70]. We confirmed the notion that *NCOA1* expression levels are affected by leptin treatment, as high leptin doses significantly upregulated *NCOA1* gene expression in MCF-7 and MDA-MB-231 cells. Moreover, *NCOA1* expression correlated positively to *LEP* and *LEPR* expression levels and STAT3 and P-STAT3 gene signatures. These findings are consistent with recent studies identifying NCOA1 as a downstream component of leptin signaling. In particular, the loss of NCOA1 in hypothalamic Pomc neurons was shown to impair leptin signaling, reducing the binding of phosphorylated STAT3 to Pomc promoters in NCOA1 KO mice [71]. In keeping with these results, NCOA1 has also been shown to enhance leptin-induced c-myc transactivation in MCF-7 BC cells [37].

Together with our results, these reports strongly support a functional interplay between NCOA1 and STAT3 within the leptin signaling pathway. Nonetheless, further investigations involving NCOA1 knockdown or STAT3 reporter assays will be required to definitively confirm the mechanistic contribution of NCOA1 to leptin-driven STAT3 transcriptional activity in BC cells.

Beyond the NCOA1/STAT3 axis, leptin can also activate PI3K/AKT and ERK/MAPK signaling cascades, which contribute to BC cell proliferation, migration, and EMT [11]. Evidence indicates that these signaling routes can interact with one another. For instance, ERK has been reported to phosphorylate and activate STAT3 [72], while phosphorylated STAT3 can, in turn, enhance MAPK/AKT signaling [65]. Moreover, crosstalk among these pathways may converge on transcriptional coactivators such as NCOA1. Supporting this, IL-6, a cytokine that shares downstream effectors with leptin, has been shown to promote NCOA1 nuclear translocation through MAPK activation [70]. Collectively, these findings highlight a complex network linking NCOA1/STAT3, PI3K/AKT, and ERK/MAPK pathways, which may cooperate to sustain the oncogenic potential of leptin. A deeper understanding of these interactions is crucial for designing combinatorial strategies to effectively disrupt leptin-driven tumor aggressiveness.

## 5. Conclusions

Taken together, our data suggest that leptin doses mimicking hyperleptinemia in individuals with obesity were able to activate NCOA1, contributing to the activation and phosphorylation of the JAK2/STAT3 pathway. This leads to BC cell proliferation, migration, and EMT. Despite these promising insights, our study has some limitations, particularly utilizing MCF-7/MDA-MB-231 cells as surrogates for real-life scenarios, thus necessitating confirmatory in vivo studies using patient-derived xenograft models or transgenic mouse models. In addition, while STAT3 was recognized as a key signaling pathway in BC progression induced by leptin, the contribution of other pathways remains viable. Moreover, our study did not address the heterogeneity within the tumor microenvironment, particularly the contribution of immune and stromal cell interactions to BC advancement. Despite these limitations, our study provides an understanding of the molecular mechanisms that drive obesity-related BC progression. Future studies validating these observations are warranted through preclinical models and examining possible pharmacological strategies.

## Data Availability

The original contributions presented in this study are included in the article/Supplementary Material. Further inquiries can be directed to the corresponding author.

## Supplementary Materials

The following supporting information can be downloaded at: https://www.mdpi.com/article/10.3390/medsci14010032/s1, Table S1: Primer sequences used for qRT-PCR.
