# Supplementary material for: Leptin Drives Breast Cancer Aggressiveness Acting Through the Activation of the NCOA1/STAT3 Pathway
Source: Med Sci (Basel). 2026 Jan 8;14(1):32. doi: 10.3390/medsci14010032 (PMC12821625; doi:10.3390/medsci14010032)
Supplement: Supplementary file 1 [file medsci-14-00032-s001.zip › medsci-3961727-supplementary.pdf]

Table S1. Primer sequences used for qRT-PCR.

Gene name	Forward primer sequence (5′–3′)	Reverse primer sequence (5′–3′)
Cyclin D1	TCTACACCGACAACTCCATCCG	TCTGGCATTTTGGAGAGGAAGTG
STAT3	CGATGCCTGTGGGAAGAG	GTCACTACGGCGGCTGTT
ERα	AGCTGTCTCCTTTCCTGCAC	GGCGTCGATTGTCAGAATTAG
ERRα	CCTGGTCTGTGGGGATGT	GGACAGCTGTACTCGATGCTC
ERRγ	GGATGGGCAAAACATATTCC	CAGAATCTCCATCCATGCAC
NCOA1	TGGCATGAACATGAGGTCAG	GCCAACATCTGAGCATTCAA
VEGF	TTGCCTTGCTGCTCTACCTCCA	GATGGCAGTAGCTGCGCTGATA
GAPDH	GGAAGGTGAAGGTCGGAGTCA	GTCATTGATGGCAACAATATCCACT
